# Obesity, physical activity, and the urban environment: public health research needs

**DOI:** 10.1186/1476-069X-5-25

**Published:** 2006-09-18

**Authors:** Russell P Lopez, H Patricia Hynes

**Affiliations:** 1 Department of Environmental Health, Boston University School of Public Health, 715 Albany Street, Talbot 2E, Boston MA 02118, USA

## Abstract

Persistent trends in overweight and obesity have resulted in a rapid research effort focused on built environment, physical activity, and overweight. Much of the focus of this research has been on the design and form of suburbs. It suggests that several features of the suburban built environment such as low densities, poor street connectivity and the lack of sidewalks are associated with decreased physical activity and an increased risk of being overweight. But compared to suburban residents, inner city populations have higher rates of obesity and inactivity despite living in neighborhoods that are dense, have excellent street connectivity and who's streets are almost universally lined with sidewalks.

We suggest that the reasons for this apparent paradox are rooted in the complex interaction of land use, infrastructure and social factors affecting inner city populations. Sometimes seemingly similar features are the result of very different processes, necessitating different policy responses to meet these challenges. For example, in suburbs, lower densities can result from government decision making that leads to restrictive zoning and land use issues. In the inner city, densities may be lowered because of abandonment and disinvestment. In the suburbs, changes in land use regulations could result in a healthier built environment. In inner cities, increasing densities will depend on reversing economic trends and investment decisions that have systematically resulted in distressed housing, abandoned buildings and vacant lots.

These varying issues need to be further studied in the context of the totality of urban environments, incorporating what has been learned from other disciplines, such as economics and sociology, as well as highlighting some of the more successful inner city policy interventions, which may provide examples for communities working to improve their health.

Certain disparities among urban and suburban populations in obesity and overweight, physical activity and research focus have emerged that are timely to address. Comparable research on the relationship of built environment and health is needed for urban, especially inner city, neighborhoods.

## Background

Persistent trends in overweight and obesity among adults and children in the United States have alarmed health care clinicians and public health practitioners. This heightened concern has yielded a rapid research effort focused strongly on the built environment, physical activity, and overweight. While much of this research is still in its infancy, certain disparities in obesity and overweight, physical activity, and research focus have emerged that are important to address.

This paper examines racial/ethnic disparities in obesity and physical activity. It then summarizes the current state of research on the built environment and health, which has been predominately suburban in focus. Next it explores the urban form – health relationship in the context of inner city environments. The paper then presents a paradox: research would predict that people of color and low income individuals would have lower obesity rates and higher physical activity rates because they live in neighborhoods that promote healthier life styles. But contrary to what theory would predict, these populations are less likely to be physically active and more likely to be obese. The paper explores the reasons for this paradox. It draws from and augments the framework of factors identified in studies of primarily suburban residential neighborhoods in order to pose a set of research questions concerning the nexus of built environment and public health in inner-city communities. We link conditions of the urban built environment to co-related social factors, including poverty, income inequality, racial segregation and economic isolation. These interlinked factors may necessitate research on more holistic and multi-sector public health policy responses and interventions in order to improve minority health impacted by the built environment of the inner city.

## Disparities in Obesity and Overweight and Physical Activity

US surveys including the Behavioral Risk Factor Surveillance System (BRFSS) and the National Health and Nutrition Examination Survey (NHANES) reveal that inner-city residents are more overweight, less physically active, and less healthy overall than the general population. Moreover, they suffer higher rates of diseases associated with obesity, namely diabetes and cardiovascular disease [1]. Data from the National Health Interview Survey (1997–1998) found that men living in center cities were more likely to be obese (39.4%) than suburban men (35.5%). Similarly, 20.6% of center city dwelling women were obese vs. 19.1% of suburban dwelling women [2]. Urban-suburban differences in physical activity were found among all adults with the urban propensity for inactivity greatest among low income people [3].

The initial flux of built environment and health research culminated in a series of articles in joint editions of the American Journal of Public Health and the American Journal of Health Promotion in 2003 [4,5]. The research hypotheses examined the role of sprawl, dependence on the car, and the design and form of suburbs on physical activity and overweight. The resultant findings were largely suburban in focus. The role of urban environment factors in overweight, physical inactivity and poor health of urban and minority residents has been less studied [6]. Contextualizing research on overweight, physical activity and the urban built environment within the reality of political, social and demographic inequality of inner cities has yet to be done.

## Built Environment and Health: What Have We Learned?

Studies on sprawl and public health have found that increased levels of sprawl are associated with increased obesity, decreased physical activity [7,8] and poorer health [9,10], including the risk of motor vehicle and pedestrian fatalities [11]. While studies on sprawl and obesity have tended to control for race, ethnicity and other individual factors, none has distinguished between effects of sprawl on inner city and suburban populations.

Evidence is mounting that the design and form of many, if not most, U.S. suburbs contribute to the growing prevalence of obesity and overweight among children and adults. Certain features of the built environment – such as the presence of sidewalks, streetlights, interconnectivity of streets, population density and use mix – appear to encourage physical activity and thus reduce the risk of obesity and related health problems. Other factors – such as cul de sacs, lack of parks, high speed traffic and automobile focused transport – may function to discourage activity and ultimately increase obesity risk [12]. Studies find that people who live close to parks are more likely to use them and to be physically active than those who live farther from them [13]. Neighborhoods with a mixture of land use types including commercial, industrial, residential and office, also appear to promote physical activity [14], while neighborhoods consisting exclusively of housing seem to dampen physical activity [15].

The built environment can affect social connectivity, motivating and stimulating interaction with others, and increasing people's trust in society and government. Alternatively, it can discourage connections with neighbors, reduce social capital, and foster a distrust of neighbors and government [16].

## An Urban-Suburban Paradox

Given a built environment in many US inner cities and urban neighborhoods that includes sidewalks and mixed land uses; that offers parks, playgrounds and public transportation; and that has a traditional gridded street pattern (small blocks with streets at right angles to each other) which fosters connectivity, we might expect that rates of physical activity and trends in obesity would be more favorable in inner city neighborhoods. But there appears instead a paradox whereby obesity, physical inactivity and associated diseases of diabetes and cardiovascular disease, are more prevalent among inner-city residents than among suburbanites. Some, if not much, of this discrepancy may be explained by other documented health risks such as the stress of poverty, racism, and violence; discrimination in health services; food insecurity; and so on. We propose that other factors of the urban built and physical environment may undermine the positive potential for being physically active in inner cities, even though urban form may, in principle, facilitate being active. We hypothesize that many factors of the environment in inner cities – including built, physical and social factors – may exert a net negative influence on the health of inner city residents and that it may be mediated in ways that are different or function differently from those in suburban neighborhoods. These factors and conditions include problem land use issues, such as waste sites; infrastructure maintenance and investment issues; and social realities, such as neighborhood crime, that can result in a neighborhood environment where outdoor exercise and recreation are risky or unappealing and are, thus, avoided (Table 1).

**Table 1:** A taxonomy of urban environmental issues

Type of issues	Representative risk factors	Potential pathways of effect
Land use issues		
	Business and job loss	Lengthens commutes, reduces walking
	Proximity of toxic facilities	Pollution may discourage outdoor activities
	Lack of supermarkets	May be more difficult to access healthy diets
	Abandoned buildings	Reduces density, increases crime
	Vacant lots	Increases crime, discourages walking
Infrastructure, maintenance and service issues		
	Sidewalks and street trees	Lack of pedestrian amenities discourages walking
	Lighting	Fear of crime keeps people indoors
	Transit projects	Lack of transit makes people more dependent on driving
Social environment issues		
	Poverty and income inequality	Poor areas suffer from disinvestment
	Racial segregation	May promote "redlining", the denial of loans to certain communities
	Crime	Unsafe areas may result in less physical activity, lower overall health status

Research on the impediments to physical activity in inner cities is needed to determine what barriers urban neighborhoods share with suburban neighborhoods and what barriers are unique to inner cities. Better understanding of the potential interaction between social and built environment factors would allow for a more fine-grained examination of the role of the built environment on the health of inner city residents. Further, research is needed to determine if a different set of design and policy responses are called for in inner cities than those aimed at reducing the obstacles to physical activity in the suburban built environment.

The issues raised in this paper are not all inclusive; nor are they always exclusive to the inner-city. They are discussed here in order to stimulate increased debate and more targeted research that may ultimately assist inner-city populations.

## Land Use Issues

The loss of commercial and industrial employers in cities has increased the physical distance between inner city residents and available jobs [17]. At one time, US metropolitan areas had strong downtown commercial districts surrounded by dense residential areas with industrial areas also close to worker housing. But decades of de-industrialization and suburbanization have resulted in a large transfer of jobs away from inner city neighborhoods towards suburban employment centers [18,19]. The result is that inner city communities are no longer close to employment opportunities, a phenomenon that economists characterize as "spatial mismatch" [20]. One consequence of this mismatch is that fewer inner city residents can walk or take local public transportation to work. They have long commutes, particularly the significant numbers that do not own cars, which ultimately could leave them little free time to be physically active or to socialize with their neighbors. To a great extent, this mismatch is not the result of zoning or government land use decisions that separate land uses in suburban areas. Rather it is the result of disinvestment and job loss in inner cities that has also contributed to the impoverishment of inner city governments and residents. Many of these economic changes have also resulted in a "loss of place" in that neighborhoods take on a tooth-gapped appearance with empty storefronts, abandoned buildings and vacant lots [21], likely making them less attractive for walking or for children playing outside.

In suburbs, solutions to the problem of special mismatch might include zoning reform and design guideline changes to allow mixed use development and encourage the development of office and commercial properties close to residential areas [22]. Inner city remedies, on the other hand, may require a different set of policy solutions, such as renewed investment in inner city neighborhoods, greater training and educational opportunities to increase skill match with existing jobs, and aggressive redevelopment of brownfields (contaminated urban parcels) for commercial/industrial development. Case studies and evaluation research of inner city neighborhood and brownfield redevelopment would help answer the question of whether and how resident physical activity is impacted by economic and neighborhood revitalization, and would garner lessons from neighborhood development models that are conducive to walking and other forms of physical exercise.

Of the non-residential land uses remaining in inner cities, many may well discourage walking and physical activity. Numerous studies have documented that hazardous waste facilities and factories that are required to report their toxic releases to the federal government (i.e., larger facilities) are disproportionately located in poor neighborhoods and communities of color [23]. An early study in Houston showed that solid waste dumps were more likely to be found in Black neighborhoods [24]. Toxic facilities in Los Angeles are more likely to be sited in minority and working class communities than in more affluent, white neighborhoods of the city [25]. A recent study of hazardous and solid waste sites, power plants, and polluting industrial facilities in Massachusetts cities and towns, found that low income communities were cumulatively exposed to environmentally hazardous facilities and sites at four times the rate of high income communities. For high minority communities, the rate was twenty times that of low minority communities [26]. Comparative studies of inner-city neighborhoods by health factors, such as exercise and prevalence of overweight, and by environmental factors, such as density and type, size, and proximity of polluting facility, would help identify the impact of local polluting facilities on residents' physical activity and overweight.

Coinciding with the health risk of physical inactivity in inner cities is the reality of food insecurity. Many older inner-city neighborhoods no longer have a local supply of fresh, healthful food and they often lack transportation access to more remote supermarkets [27]. Supermarkets are less likely to locate in inner cities and small stores are more likely to sell low quality, non-fresh food and have higher prices, a situation that would contribute to poorer nutrition and lower health status [28]. The separation of residential areas from commercial services and goods, such as large supermarkets in inner cities, is not the result of intentional zoning policy as in suburbs; rather it is a consequence of social and economic trends, including business flight, that have impoverished older urban neighborhoods. Studies of diet and nutrition among inner city residents need to include research on the prevalence of, proximity to, and transportation access to large supermarkets, as well as comparisons of price and food quality between urban and suburban food markets.

Decades of racial change, segregation, economic disinvestment and discriminatory lending and insurance practices have resulted in a severe reduction of the housing stock in many urban communities, often accompanied by new construction in distant areas [29]. The social effect of abandonment has been termed "the broken window syndrome": Neighborhoods with broken windows and dilapidated housing encourage crime, pose safety hazards, isolate residents and reduce trust in the ability of the neighborhood to meet its challenges [30]. One study has linked these factors of neighborhood quality to the risk of acquiring sexually transmitted diseases [31]. Another consequence of this constellation of risks from a moldering environment would likely be reduced walking, physical activity and recreation in public.

Comparative studies of building conditions, rates of children's play out-of-doors and resident walking could reveal a relationship between residential conditions and physical activity. Poor built environment conditions beg the research question: What is the relationship between building condition, building tenancy, building abandonment and rates of physical activity and obesity?

Even when abandoned buildings are demolished, the resulting vacant land poses problems to the environment [32]. If untended, these lots usually become overgrown with weeds and covered with litter, an invitation for illegal dumping (including demolition and construction debris, hazardous chemicals and medical wastes), and foster criminal activity. Does vacancy contribute to less walking, playing, and other physical activity in the affected neighborhood? This question, like its companion regarding abandoned housing, has yet to be examined.

If low density is a factor implicated in physical inactivity as some have posited it is important for policy implications to distinguish between lower density neighborhoods caused by abandonment and those resulting from zoning and land use decisions. While the latter is the intentional result of government decision-making, the former is a consequence of a more complex mixture of forces that include public and private sector lending and insurance practices as well as social relations among races in the United States. Solutions, which have centered around increased investment, elimination of discriminatory lending practices and the creation of new, beneficial land uses in inner city communities, such as side lot disposition programs, community gardens, and housing rehabilitation [33], ought to be evaluated for their potential impact on multiple forms of physical activity, such as more time spent outdoors in walking, gardening, biking, and so on.

## Infrastructure, maintenance and service issues

The social context of the inner city environment, specifically inequity, can affect the quality of infrastructure and how new improvements are planned and implemented [34]. standardized tests over time for recreation, is resulting in less gym and recess in the school day. Cyclic decay in urban parks and playgrounds has rendered some of them un-useable and dangerous havens for criminal activity. The net effect is to reduce the utility of local recreational facilities for many inner city residents and reduce opportunities for social interaction. These factors will only be addressed by a change in government priority and investment and by social mobilizing to preserve, maintain and enhance physical activity opportunities. Evaluation research is needed on the community health benefits of restoring degraded parks and playgrounds with regular recreational opportunities for children and adults in order to support the lobby for more public investment in parks and playgrounds.

The authors are familiar with programs that have restored inner city schoolyard environments. The Boston Schoolyard Initiative (BSI) is a creative example of private-public partnership that has restored 60 Boston public school playgrounds for recreation and play using community collaborative design [35]. While none of the renovated schoolyards has been evaluated for change in physical activity by amount or kind, the BSI does intend to measure activity change in students using a set of validated questionnaires and observational tools.

A Harlem Hospital physician who documented the increase in child injury on debilitated school and parks department playgrounds and a gardener for the New York City Department of Parks and Recreation developed a collaborative venture called The Greening of Harlem. They renovated numerous school playgrounds, turning them from cracked and jagged concrete and blacktop surfaces with broken metal play equipment into safe and creative performance and play spaces with vegetable and flower gardens [36]. Evaluation of inner-city interventions is often sacrificed because scarce resources are spent on projects and programs that are desperately needed and inherently challenging to accomplish.

A related series of observational studies of the built and natural environment in Chicago public housing, one of the starkest of urban built environments, found that greater numbers of people gathered in outdoor spaces with trees than in those without trees [37], the distance to each being equal. A companion study of Chicago public housing play space found that children in highly vegetated spaces played more (by a factor of two) than children in non-vegetated areas, and that they played more creatively and interacted more with adults [38]. These studies raise the question of the role and value of trees, vegetation and nearby nature in inner cities in promoting physical activity and play. Is their presence sufficient? Are factors such as their condition and maintenance and their proportion to the recreational space important in their effect of promoting play and activity?

Over decades, unimproved sidewalks decay as utility crews dig up concrete, tree roots push up paved areas, and weather erodes surfaces. Many cities lack the resources to repave or replace sidewalks as frequently as needed; and many urban neighborhoods lack the political clout to have their sidewalks prioritized for repair or reconstruction. As a result, urban neighborhoods frequently have broken or impassable pedestrian sidewalks. While sharply contrasting with the problem of no sidewalks in many suburban communities, broken sidewalks in inner cities likely have a similar outcome, less leisure walking [39]. Sidewalk replacement and repair in city neighborhoods provides an opportunity to study the question of whether passable sidewalks result in more local walking.

Street trees, along with other pedestrian amenities, have been found to be a promoting factor in physical activity [40]. In many urban areas, street canopies are disappearing as disease, age, and lack of maintenance of new trees, slowly reduce the number of trees. When cities suffer fiscal constraints, the budgets of parks and recreation departments are generally the first to be cut; and replacement of urban street becomes a low priority. Further, evidence suggests that trees are more likely to be replaced in higher income communities than in neighborhoods with more poor households and people of color [41].

Solutions to the decay of street and sidewalk infrastructure depend on improving the fiscal environment of inner cities [42]. Unless center cities have access to adequate capital and operating funds, the physical environment will continue to decay. This decline in infrastructure is more likely to be concentrated in poor and minority neighborhoods and has been documented for a wide range of services including fire stations and hospitals [43,44].

Traffic lights can both improve and inhibit pedestrian activity. When they are well timed and placed to reduce traffic flows and improve pedestrian safety, they may encourage physical activity and social interaction. But when they are timed to improve traffic speeds or are missing, they can discourage pedestrian activity [45]. Bus shelters, public transportation and "traffic calming" can be a net boon to pedestrian activity. Conversely, intersections engineered for cars, increased speeds in residential and commercial areas, and even large parking lots can result in local people spending less time outdoors and doing less physical activity [46]. Older cities are increasingly being redeveloped with the same engineering standards of newer, automobile focused suburbs rather than in keeping with older, pedestrian-oriented communities. The new roads and parking facilities do not promote physical activity in the way older streets once did [47]. Policy research on redevelopment in inner cities could examine and reconcile the transportation design in inner city re-development with emerging pedestrian-friendly design guidelines for suburban development.

Transportation improvements, like other types of infrastructure investments, take place in the context of political and social decision making. To the extent that poor inner city neighborhoods lack the ability to make their transportation needs a political priority, they can suffer from a decline in services or a lack of access to new or existing improvements. In Los Angeles, the Metropolitan Transit Authority shifted investments to commuter rail and large subway lines that served more affluent communities to the detriment of the buses that had traditionally provided services to lower income neighborhoods [48]. In Boston, new suburban rail lines that will serve up to 5000 passengers per day have received more funding than new public transport for inner city communities designed to serve 50,000, even when investments were promised and owed to inner city residents as replacement for the 20 year loss of a pre-existing subway [49]. Increasing transportation options for inner city residents, who are often the most transit dependent population and less likely than suburbanites to own cars, needs to be made at least as high a priority as suburban transportation investment. Research on the impacts of local and regional transportation investments on physical activity and health will add evidence to support more equitable transportation policy.

Federal highway construction that mushroomed together with the suburban housing boom after World War II bifurcated many older communities and devastated others. In numerous metropolitan areas, neighborhoods with large numbers of minorities, poor and working class were specifically targeted for new highways that connected expanding suburbs with existing downtowns. Neighborhood businesses and affordable housing were razed by state land taking under the power of eminent domain; and the new highways functioned as physical barriers within neighborhoods for those without a car. Miami's Overtown neighborhood was devastated – churches demolished, commercial districts destroyed and residential areas literally severed from each other – by the construction of Interstate 95 [50]. Similarly, New Orleans's black community suffered from highway construction with neighborhood institutions destroyed, affordable housing lost and residents displaced [51]. Wherever cities make major transportation infrastructure changes, studies of local populations are vital to document the impact on their physical mobility and activity, their stress level and quality of life.

## Social Environment Issues

Both low income and increased income inequality have been found to be associated with a decrease in physical activity and an increased likelihood of poor health [52,53]. Living in economic isolation, that is a neighborhood with a high percentage of low income people, has also been found to be a risk factor for poor health [54]. People with low incomes likely have less time to be physically active because they are working multiple jobs, and because they are more likely to be concentrated in neighborhoods without the amenities (or with deteriorated amenities) found in more affluent communities [55]. Likewise, income inequality, which has been increasing since the early 1970s [56], may result in poor communities having fewer recreational resources and ones of lower quality than would be the case where income inequality is less extreme. Assessing and addressing the burdens of inequality, which fall most heavily on the poor and near poor, can only help to improve the health of inner city residents. Results can help identify underserved neighborhoods and prioritizing them for physical activity intervention programs.

Residential racial segregation, the physical separation of people based on skin color or ethnic origin, has declined slightly from its peak in the 1950s but continues to be a substantial problem in many metropolitan areas [57]. Interestingly, the greater the Black-White segregation, the more likely all residents, both Black and White, are to be physically inactive or otherwise unhealthy [58]. Segregation has also been found to influence the impact of infectious diseases, reducing overall health status and further decreasing physical activity levels [59]. Segregation has also been linked to increased air pollution, potentially increasing risk to cardiovascular and respiratory health [60]. Higher levels of segregation are linked with increased exposure to violence, less time outdoors and an overall increase in the allostatic load, that is the sum of ill health, as a result of increased stress [61]. Increased stress, when sustained over time, is known to have immune related consequences and to increase the risk of cardiovascular disease and other health problems [62]. Additional research is needed regarding the impact of segregation on rates of physical activity, obesity and access to recreational resources and nutritious food.

Crime and the fear of crime are a reality in many inner city communities. Crime erodes community trust, marginalizes residents, and further stresses people and the environment [63]. Fear of crime is likely to keep people indoors, particularly the old and the young, and discourage physical activity. Results from a recent asthma research project in one of Boston's most distressed public housing developments, which had also experienced a series of murders in or near the development, revealed that 63 % of those interviewed were afraid of violence in the neighborhood and 60% did not allow their children to play outside because of neighborhood violence. This was a significantly higher response when compared with residents of housing developments in other neighborhoods, which had not experienced the wave of violent crime [64].

Increasing physical activity among inner city people may hinge significantly on increasing people's sense of neighborhood security. Many research questions arise here: Does change in local crime rates affect change in rates of local physical activity? Do positive neighborhood activities, such as community gardens, park cleaning, and crime watch, result in changed rates of crime, leisure walking, socializing, playing outdoors, and more walking trips to local destination points such as library, stores, and so on?

## Discussion

Given the overall burden of poverty, discrimination and prevalence of crime in many older inner-city neighborhoods, it is no surprise that health status is lower in these communities. Built environment also may play a role in poor health, by discouraging physical activity, recreation, and social interaction and by limiting access to nutritious food. Problems in the built environment may interact with and compound other social and health issues and thus magnify the community health effects [65]. The challenge is to identify the nature and extent of the activity- and health-related built environment factors in inner-city neighborhoods – many of which have been studied in suburbs – and also to create new measures of the built environment that incorporate the unique social and physical characteristics of urban inner city environments [66].

Improving the built environment in inner cities was crucial before the recent public health awareness of the correlation between built environment and obesity with its health-related diseases. However, policy initiatives that reduce barriers to physical activity in middle class and affluent suburbs do not assuredly translate to improvements in inner city health [67]. Some policies, such as better designs that encourage mixed use development and traffic calming [68], may be applicable in both suburb and city, especially in the context of urban re-development. In fact, these design policies prevailed in original inner city master plans. However, other needed interventions are unique to urban cores. These include: investment in inner city development that also builds social capital, prioritizing brownfield redevelopment, renewing efforts to reduce racial segregation and economic isolation, and addressing the trend in income inequality in cities [69]. Government policies on urban renewal and redevelopment have had the historical effect of displacing and isolating people of color and reducing their ability to move to healthier communities, a consequence of which is severe health disparities between people of color and others [70, 71].

Studies of the impact of sprawl on inner cities need to be designed to identify specific effects on urban form and resident physical activity, as has been done for metropolitan areas and suburbs [72,73]. In addition, community-based initiatives that can incorporate the energy and experience of inner city residents, and the special knowledge of community based institutions are needed to assure that interventions and policy initiatives are effective in identifying solutions to the epidemic of obesity and related chronic disease in inner cities [74,75].

Effective research on urban areas must involve the people and communities being studied. Community-based Participatory Research (CBPR) provides a framework for researchers interested in the urban built environment. Briefly, the CBPR principles that should guide research include: neighborhoods should have input into the types of issues being studied as well as study design; researchers should take care to communicate the results of these studies back to the neighborhoods where the research took place; and studied communities should benefit from research [76]. Using the expertise of residents can energize research and neighborhood change. Research on inner city communities has sometimes been seen as problematic in these neighborhoods with problems ranging from inappropriate burdens on time placed on already overburdened individuals and institutions to research that bypasses local residents all together. Consultation with local residents can help researchers better target their research and help find the right balance between involvement and resource use.

Issues of scale and neighborhood definition complicate efforts to research and intervene on the urban environment. Neighborhood definitions can be fluid, and even a given individual's concept of neighborhood can vary over time, place or issue. Furthermore, neighborhoods do not exist in isolation but are subunits of larger city, county, metropolitan, state, national or even transnational entities. Given the enormous potential impact of these other scales, it is important to study the effects of neighborhood in context. In practice, this may necessitate using multilevel modeling or other techniques to fully capture local and regional effects. In any case, researchers should be explicit on their definitions of neighborhood and clearly state the type of urban/suburban environment that is being studied.

It is also important to remember that the current conditions in a neighborhood reflect past policies, conditions and events. Individuals and institutions sometimes carry a memory of the past and sometimes neighborhoods undergoing demographic change may no longer carry an understanding of history. As part of the process of studying or improving communities, researchers can help neighborhoods learn the historical precedents of contemporary environments as well as utilize the knowledge of current residents, particularly elders, to develop a more thorough understanding of a neighborhood's history. Gathering oral histories, searches of archival databases (including public records), focus groups, or detailed questionnaires targeting key individuals can assist in these efforts.

Studies of built environment – health interactions represent a reconnection of the intersection of public health and urban planning. But these are not the only disciplines that should be included in this line of research. To fully utilize the potential of the built environment to better the health of urban communities, it will be necessary to consult economists, sociologists, anthropologists, education specialists, among others. Landscape architects, urban historians, traffic engineers and botanists all should be involved in the effort to study and improve urban environments. Multidisciplinary teams and transdisciplinary research are best for addressing the multi-dimensional issues confronting our urban areas.

## Conclusion

Recent research on the health impacts of the built environment has led to a better understanding of how contemporary land use planning may influence physical activity, obesity and related chronic diseases. Much of the focus of this research has been on suburban development design and form. Comparable research on the relationship of the built environment and health is needed for urban, especially inner city, neighborhoods. The research should be designed to include the web of social and political factors that contribute to the conditions of the built environment of inner cities, if the consequent solutions and policy initiatives to increase physical activity and promote health are to succeed.

## Competing interests

The author(s) declare that they have no competing interests.

## Authors' contributions

Both authors helped to draft the manuscript. Both authors read and approved the final manuscript.
